# Efficacy of Colchicine and Budesonide in Improvement Outcomes of Patients with Coronavirus Infection 2019 in Damascus, Syria: A Randomized Control Trial

**DOI:** 10.1155/2021/2129006

**Published:** 2021-12-31

**Authors:** Mohammad Alsultan, Ameer Obeid, Omar Alsamarrai, Mohamed Taher Anan, Aliaa Bakr, Nawwar Soliman, Mamdoh Kurdy, Muhannad Hag Mosa, Zain Saleh, Fatima Hujij, Jafar Barhoum

**Affiliations:** ^1^Department of Nephrology, Al Assad and Al Mouwasat University Hospitals, Damascus, Syria; ^2^Department of Infectious Diseases, Al Assad and Al Mouwasat University Hospitals, Damascus, Syria; ^3^Department of Neurology, Al Assad and Al Mouwasat University Hospitals, Damascus, Syria; ^4^Professor, Statics Department, Aleppo University, Aleppo, Syria; ^5^Department of Oncology, Al Biruni University Hospital, Damascus, Syria; ^6^Department of Internal Medicine, Al Assad and Al Mouwasat University Hospitals, Damascus, Syria; ^7^Department of Rheumatology, Al Assad and Al Mouwasat University Hospitals, Damascus, Syria

## Abstract

COVID-19 was reported in China in 2019 and has spread worldwide. Transmission occurs through respiratory secretions and, less commonly, through contaminated surfaces. The severity of the disease can range from asymptomatic to acute respiratory distress syndrome (ARDS). In this study, we aim to investigate the efficacy of two agents (oral colchicine and budesonide inhaler) in COVID-19 infection management, compared with supportive care alone. 77 patients were admitted to the isolation section of Al Assad University Hospital, between the 1^st^ of August and the 30^th^ of August. A total of 49 patients were included in this randomized control trial, after excluding ineligible patients. The random sample was divided into three groups; the first group was supportive care plus colchicine, the second group was supportive care plus budesonide inhaler, and the control group was supportive care alone. PaO2/FiO2 was improved in the budesonide group, higher than the supportive and colchicine groups. The median hospitalization days were shorter when using colchicine or budesonide, opposed to supportive care alone (8 vs 10 days, respectively). 34 patients (69.3%) were discharged, and 27 patients (55.1%) were followed up until they were weaned from oxygen and made a complete recovery. There was a significant decrease in mortality with colchicine (3 patients; 21.4%) compared with supportive care (7 patients; 33.3%) and the budesonide group (5 patients; 35.7%).

## 1. Introduction

COVID-19 was reported in China in 2019 and has spread worldwide. Transmission occurs through respiratory secretions and, less commonly, through contaminated surfaces [1]. The severity of disease can range from asymptomatic to acute respiratory distress syndrome (ARDS). 81% of cases were defined mild if no pneumonia or mild pneumonia was presented; 14% were defined severe when SpO2 ≤93%, respiratory rate ≥30 breaths/min, PaO2/FiO2 <300 mmHg, and/or lung infiltrates >50% within 24 to 48 hours; and 5% were defined critical if septic shock, respiratory failure, and/or multiple organ dysfunction occur [2]. Death and hospitalization in patients with medical conditions (19.5% and 45.4%, respectively) were higher than those without medical conditions (1.6% and 7.6%, respectively) [3]. Other study reported a 49% fatality rate in critical cases [4].

### 1.1. Pathophysiology of ARDS

ARDS was divided into three phases: exudative, proliferative, and fibrotic [5]. Increased permeability and protein-rich pulmonary edema is the hallmark of disease [6]. Several potential mechanisms were implicated; neutrophil accumulation was consistently seen in the early phase of ARDS, which releases several proteolytic enzymes, cytokines (TNF-*α*, IL-1*β*, IL-6) [7], and reactive oxygen species [8].

The proliferative phase is associated with a shift from neutrophils to lymphocytes, proliferation of type II pneumocytes, and a marked increase of type III procollagen peptides, which is associated with increased mortality [5].

The third phase of fibrotic injury is called fibrosing alveolitis, resulting from the interaction of fibroblasts, epithelial cells, cytokines (IL-1), and growth factors [9, 10]. Interstitial fibrosis, acinar disruption, and emphysema-like changes are consequences of this phase [5].

### 1.2. Pharmacology

Various drugs (antiviral and anti-inflammatory agents) are currently being investigated for COVID-19 treatment, and current recommendations support the efficacy of remdesivir and dexamethasone to treat critically ill patients (hospitalized and required supplemental oxygen) [1].

Colchicine arrests microtubule formation and elongation, which are involved in cellular processes, as maintenance of cell shape and cell migration [11]. This inhibits adhesiveness and lysosomes degranulation and motility [12] and mitigates inflammatory cytokines, such as interleukin (IL-1, IL-6), and tumor necrosis factor (TNF)-*α* [13]. The antifibrotic effect of colchicine is shown by inhibiting myofibroblast differentiation, suppressed smooth muscle cell proliferation, and vascular endothelial growth factor (VEGF) with decrease cell apoptosis [14–16].

Budesonide is a nebulized glucocorticoid used to treat chronic obstructive pulmonary disease (COPD) and asthma. Budesonide causes significantly decreasing levels of adhesion molecules such as ICAM-1 and MIP-2, which are released from injured epithelial and endothelial cells causing macrophages and neutrophil infiltration [17, 18]. Budesonide can decrease proinflammatory cytokines (TNF-*α*, IL-1*β*, IL-6) [19, 20], increase the IL-10 levels, which can antagonize the effects of previous cytokines [21], and reduce cell apoptosis of the lung [22]. Budesonide reduces edema, alveolar wall thickening, hyaline membrane formation, and lung tissue damage [23].

In this study, we aim to investigate the efficacy of two agents (oral colchicine and budesonide inhaler) in COVID-19 infection management compared with supportive care alone.

## 2. Materials and Methods

### 2.1. Patients

A total of 77 patients were admitted to the isolation section of Al Assad University Hospital between August 1 and 30. 49 patients were included in this randomized control trail by randomized number tables after excluding ineligible patients. Admission criteria for all patients were oxygen saturation ≤93% plus at least one of the following: (1) respiratory rate ≥30 breaths/min, (2) infiltrates >50% on CT scan, and (3) arterial partial pressure of oxygen (PaO2)/fraction of inspired oxygen ratio (FiO2) <300 mmHg. The inclusion criteria were as follows [24]: (1) adult (aged ≥18 years), (2) patients with positive PCR test of COVID-19 virus in specimens taken from the respiratory tracts, and (3) patients with negative PCR test but had clinical signs and symptoms of viral illness accompanied with chest CT scan showing the radiologic findings of viral pneumonia, which was defined as new, unexplained, and bilateral infiltrates on the lungs. Exclusion criteria were patients who (1) were admitted for other conditions with oxygen saturation ≥94% without viral symptoms but had infiltrations on chest CT scan (mild form of COVID-19) [25], (2) received other antiviral or investigational therapies for COVID-19 [24], (3) expired or transmitted to ICU during the first 24 hours [26, 27], and (4) patients who committed with persistent treatment of steroid inhalers [28].

### 2.2. Procedures

The random sample divided into three groups: first one was supportive care plus colchicine (oral colchicine 1.5 mg followed by 0.5 mg after hour in day 1, then 0.5 mg twice daily for the next 4 days) [27], second one was supportive care plus budesonide inhaler (200 mcg twice daily for 5 days in an inhalation chamber), and the control group (third one) was supportive care only. All patients received appropriate supportive care with oxygen supplementation, vitamins, anticoagulants, dexamethasone, prone position, noninvasive ventilation (CPAP or BIPAP), antibiotics, and fluids. Vitamins consist of vitamin C, vitamin D, and zinc. All patients had taken anticoagulants according to Guideline Thromboprophylaxis-and-Anticoagulation-in-COVID-19-infections [29]. Dexamethasone was given 4 mg twice daily for 10 days. Conservative fluid management was given if patients had acute kidney injury (AKI) or hemodynamic instability and stopped when serum creatinine returns to baseline or improved vital signs [30]. Prone position ≥12 hours/day was used for tolerant patients [31]. Noninvasive ventilation (NIV) was used for tolerant patients [32]. All patients received a treatment for severe community-acquired pneumonia (CAP), which consists of azithromycin plus ceftriaxone or levofloxacin for 5 days. Levofloxacin was used as an initial antibiotic if patients were on colchicine or previously took azithromycin. Other broad-spectrum antibiotics were used if patients developed symptoms of hospital-acquired pneumonia (HAP). All antibiotics were stopped after 5 days with normal limits of procalcitonin. When patients were transmitted to the ICU, the studied drugs were discontinued and the patients were followed up to report the outcome.

Infiltrations were estimated with chest CT in the first day and reevaluated if patients clinically deteriorated. Oxygen saturation was checked three times daily during admission using a pulse oximeter device. Laboratory tests include arterial blood gases (ABGs), CPK (creatine phosphokinase), LDH (lactate dehydrogenase), electrolytes, liver function tests (LFTs), kidney function tests (KFTs), CRP (C-reactive protein), and hematologic tests. Procalcitonin (pro-CT) was tested within 5 days of admission and reassessed if HAP was clinically suspected. Patients were transmitted to the ICU based of available beds and clinically deterioration despite a trail of noninvasive ventilation. All patients were discharged after ABGs showed oxygen saturation (SpO2) ≥92% on air room or nasal cannula and respiratory rate (RR) <30 breaths/min. We followed up the discharged patients via serial phone calls until full recovery and oxygen discontinuation.

### 2.3. Ethical Consideration

Written or verbal inform consent for inclusion was obtained from all participants before they participated in the study. The study was conducted in accordance with the Declaration of Helsinki, and the protocol was approved by the Ethics Committee of Damascus University.

### 2.4. Statistical Analysis

Analysis was performed using the statistical programs SPSS version 20 and *R* 4.02. We also used nonparametric statistics such as chi-square test and parametric statistics such as paired-samples *T*-test and one-way analysis of variance (ANOVA). Descriptive analysis was performed using arithmetic mean and standard deviation. *P* value < 0.05 was considered statistically significant.

## 3. Results

This study was conducted on 49 patients admitted to Al Assad University Hospital. Every patient underwent a single COVID-19 PCR test. 31 patients had positive COVID-19 PCR results, and 18 patients had negative results but had clinical signs and symptoms of viral illness along with radiologic findings on chest CT compatible with COVID-19 infection.

In total, 19 males and 30 females were analyzed, divided into three groups: 21 patients in the supportive group, 14 patients in the colchicine group, and 14 patients in the budesonide group. There were no statistically differences between three groups in age, gender, and comorbidities (Table 1). Most patients (31 patients) were obese with body mass index (BMI) ≥30 kg/m^2^. History of hypertension and diabetes was observed in 23 and 26 patients, respectively.

On admission, all patients had no differences on admission criteria, laboratory findings, creatinine clearance (Crcl), Padua prediction score, supportive therapies, and complications of COVID-19 (Table 2). 33 patients were admitted with oxygen saturation ≤93% with two or more admission criteria. 24 patients had WBC (white blood cell) count between 10,000 and 20,000/ml, and 47 patients had lymphocyte percentage less than 20%. 30 patients had creatinine clearance (Crcl) ≥60 ml/min/1.73 m^2^, and 27 of all patients had normal limits of creatinine (Cr) (0.5–1.2 mg/dl); however, 30 patients had urea (Ur) levels ≥45 mg/dl. 33 patients had normal alanine aminotransferase (ALT) levels (≤41 mg/dl); however, a slight elevation (38–100 mg/dl) in aspartate aminotransferase (AST) was seen in 24 patients. Creatine kinase (CK) was within normal limits (≤170 mg/dl) in 38 patients, but lactate dehydrogenase (LDH) was elevated (≥480 mg/dl) in 43 patients. C-reactive protein (CRP) was elevated (≥0.5 mg/dl) in all patients; up to 10 mg/dl in 23 patients and other 26 patients with CRP ≥10 mg/dl; however, procalcitonin (pro-CT) levels were normal (less than 0.5 mg/dl) in 38 patients. Half patients had D-dimer ≥1000 ng/ml.

All patients scored ≥4 on the Padua prediction score. Antibiotics were prescribed as azithromycin alone (7 patients), azithromycin plus ceftriaxone (24 patients), levofloxacin alone (16 patients), and other broad-spectrum antibiotics (2 patients). For anticoagulants, 20 patients received enoxaparin 40 mg/SC/qDay, 7 patients received apixaban 2.5 mg/PO/bid, and other 22 patients had apixaban 5 mg/PO/bid.

Oxygen saturation between admission and discharge was analyzed in 34 patients, and there was no statistically differences (Table 3), but the comparison was analyzed between admission-saturation on devices (cannula, face mask, or non-rebreather mask) and discharge-saturation on air room or nasal cannula, which means mitigated deterioration and significant improvement in oxygen demands in three groups.

On admission, a PaO2/FiO2 ratio of ≤100 mmHg consistent with severe ARDS was observed in 35 patients (71.4%), and 10 patients (20%) had a PaO2/FiO2 ratio between 100 and 150 mmHg. The PaO2/FiO2 ratio between admission and discharge was studied in 25 patients and showed statistically significant improvement in all groups (Table 3). PaO2/FiO2 was improved in the budesonide group (from 80 to 217, *P* ≤ 0.01), higher than supportive (from 102 to 221, *P* ≤ 0.016) and colchicine groups (from 118.4 to 200, *P* ≤ 0.001) (Table 3).

The median hospitalization days for groups with colchicine or budesonide was shorter than the group with supportive care only (8 vs 10 days, respectively), but with no statistically significant differences (*P* = 0.60) (Table 4). 27 patients were followed up until weaning from oxygen, and the median days on oxygen supplementation (from the day of admission to the day they stopped using oxygen) were 20 days in the supportive group, 19 days in the colchicine group, and 20 days in the budesonide group (*P* = 0.97) (Table 4).

34 patients (69.3%) were discharged, 27 patients (55.1%) were followed up until weaning from oxygen and complete recovery, and 6 patients (12.2%) had been readmitted due to other conditions. The remaining 15 patients (30.6%) were transferred to the ICU and died later. Mortality was decreased in the colchicine group (3 patients, 21.4%) compared with supportive care (7 patients, 33.3%) and budesonide groups (5 patients, 35.7%) (Table 4).

## 4. Discussion

In this randomized controlled trial, 5-day treatment of oral colchicine or budesonide inhaler mitigated respiratory deterioration and decreased hospital length in moderate to severe ARDS patients. Moreover, reduced mortality was observed by using colchicine compared with placebo.

Both colchicine and budesonide reduced the mean time of hospitalization compared with supportive care only (8 vs 10 days, respectively), and this is clinically important in COVID-19 pandemic due to reducing the hospital length and the need for hospital beds. This corresponds with a study that showed reducing the length of hospitalization by 2.5 days in patients receiving colchicine [25].

Other RCT showed the clinical recovery was 1 day shorter in the inhaled budesonide arm compared with the usual care arm [33].

Budesonide improved oxygen saturation and PaO2/FiO2 ratio but had no benefit (*P* = 0.67) on mortality compared with supportive care (35.7% vs 33.3%). This corresponded with studies that used different budesonide doses (1 mg/twice daily) [23, 28]. However, low-dose (200 mcg/twice daily) and short-term treatment of budesonide in this study reduced oxygen demands and consequently reduced costs.

Colchicine treatment reduced mortality (21.4%) compared with supportive care alone (33.3%) and additionally improved oxygen saturation and PaO2/FiO2 ratio. Similar results were reported in previous studies [25, 27, 34]. Although, one study used a higher dose of colchicine, one patient was admitted to the ICU, explaining that colchicine would not prevent respiratory failure for every patient with severe ARDS (SatO2/FiO2 ≤100 mmHg) [25]. The rest studies used a longer course of colchicine [27, 34]. In this study, 5-day treatment of colchicine reduced mortality, although most patients were classified as severe ARDS.

Overall mortality was 15 patients (30.6%), higher than reported in a study in United States [3]. However, most patients in this study had several comorbidities and worse clinical status on admission, which is observed by PaO2/FiO2 ratio ≤100 mmHg in 35 patients (71.4%) and low values of mean PaO2/FiO2 ratio compared with higher PaO2/FiO2 ratio in previous studies [23, 25, 27, 34].

In spite of discontinuation after ICU transmission and short-course treatment, using budesonide and colchicine alleviated clinical deterioration and reduced hospital length; moreover, mortality was reduced by using colchicine. Low cost and global availability of colchicine and budesonide make them a choice for treating COVID-19. This study had some limitations, such as lack of blinding and reduced number of participants in a single isolation ward.

## 5. Conclusion

Using colchicine and budesonide in moderate to severe ARDS patients showed better evolution of disease, which is observed by reduced hospital length and respiratory deterioration in addition to reduced mortality with colchicine. Evaluation of these drugs on ARDS induced by COVID-19 may require an early employing and evaluation therapy in ventilated patients.

## Figures and Tables

**Table 1:** Demographics and clinical characteristics.

Demographic/comorbidities		Supportive	Colchicine	Budesonide	*P* value
Age (mean), years	18–40	ـــــ	ـــــ	ـــــ	ـــــ
	40–60	53	50	55	0.70
	60–80	69	60	67	0.67
	≥80	81	89	80	≤0.01
Gender	Male	9	5	5	0.87
	Female	12	9	9	0.87
Smoking	Nonsmoking	14	8	11	0.48
	Smoker	7	6	3	0.48
BMI	<30	8	5	5	0.98
	≥30	13	9	9	0.98
Kidney disease	None	10	10	10	0.06
	CKD	2	1	1	0.96
	AKI-1	3	2	1	0.79
	AKI-2	1	0	2	0.27
	AKI-3	1	1	0	0.62
	AKI on CKD	4	0	0	0.03
Cardiac disease	None	7	5	6	0.84
	HTN	10	6	7	0.92
	CHF	ـــــ	ـــــ	ـــــ	ـــــ
	Arrhythmia	0	0	1	0.14
	CAD	ـــــ	ـــــ	ـــــ	ـــــ
	Others	4	2	0	0.25
Endocrine disease	None	7	7	6	0.27
	Diabetes	14	7	5	0.25
	Others	0	0	3	0.09
Neurologic disease	None	15	12	12	0.47
	Stroke	2	0	0	0.36
	Others	4	2	2	0.32
Other diseases		2	4	0	0.06

**Table 2:** Admission criteria, laboratories, and additional supportive treatments.

Laboratory characteristics		Supportive	Colchicine	Budesonide	*P* value
Admission criteria	sat ≤ 93% + 1 condition	4	4	0	0.11
	sat ≤ 93% + 2 conditions	9	4	6	0.69
	sat ≤ 93% + 3 conditions	8	6	8	0.70
WBCs (white blood cells)	≤10th/ml	8	7	7	0.70
	10–20th/ml	13	7	4	0.21
	≥20th/ml	0	0	3	0.01
Lymphocytes	≤5%	9	1	5	0.05
	5–10%	6	6	4	0.36
	10–20%	6	6	4	0.62
	≥20%	0	1	1	0.46
HB (hemoglobin)	≤10 g/dl	6	4	1	0.26
	10–13 g/dl	9	5	10	0.12
	≥13 g/dl	6	5	3	0.87
PLT (platelets)	<150th/ml	5	1	3	0.40
	≥150th/ml	16	13	11	0.40
Ur (urea)	≤45 mg/dl	8	6	5	0.02
	45–100 mg/dl	6	5	5	0.87
	≥100 mg/dl	6	3	5	0.74
Cr (creatinine)	0.5–1.2 mg/dl	11	8	8	0.94
	1.2–2 mg/dl	4	3	2	0.83
	2–3 mg/dl	3	2	3	0.83
	≥3 mg/dl	3	1	1	0.78
ALT (alanine aminotransferase)	≤41 mg/dl	15	9	9	0.87
	41–100 mg/dl	5	5	5	0.87
	≥100 mg/dl	1	0	0	0.51
AST (aspartate aminotransferase)	≤38 mg/dl	11	5	6	0.61
	38–100 mg/dl	8	9	7	0.31
	≥100 mg/dl	2	0	1	0.44
CK (creatine kinase)	≤170 mg/dl	17	9	12	0.35
	170–500 mg/dl	4	4	0	0.11
	≥500 mg/dl	0	1	2	0.27
LDH (lactate dehydrogenase)	≤480 mg/dl	5	0	1	0.08
	480–1000 mg/dl	9	8	10	0.25
	≥1000 mg/dl	7	6	3	0.48
PT (prothrombin time)	<60%	4	0	3	0.19
	≥60%	17	14	11	0.19
INR (international normalized ratio)	≤1.2	16	12	10	0.65
	>1.2	5	2	4	0.65
CRP (C-reactive protein)	≤0.5	ـــــ	ـــــ	ـــــ	ـــــ
	0.5–10	9	9	5	0.28
	10–20	7	4	5	0.91
	≥20	5	1	4	0.08
Pro-CT (procalcitonin)	<0.1	8	5	5	0.27
	0.1–0.5	8	7	5	0.70
	≥0.5	5	2	4	0.65
D-dimer	<1000	12	8	4	0.19
	≥1000	9	6	10	0.19
Crcl (creatinine clearance)	≥90	7	5	5	0.96
	90–60	4	5	4	0.53
	60–30	6	3	3	0.84
	<30	4	1	2	0.17
Antibiotic	Azithro	3	3	1	0.36
	Azithro + ceftriaxone	11	7	6	0.85
	Levofloxacin	6	4	6	0.63
	Others	1	0	1	0.62
Padua score	<4	ـــــ	ـــــ	ـــــ	ـــــ
	≥4	21	14	14	1
Anticoagulants	Enoxaparin 40 mg/d	9	5	6	0.90
	Apixaban 2.5 mg/twice/d	3	3	1	0.56
	Apixaban 5 mg/twice/d	9	6	7	0.93
Other drugs		10	6	4	0.52
Complications of COVID-19		6	4	6	0.62

**Table 3:** Oxygen saturation and PaO2/FiO2 ratio between admission and discharge.

Study outcomes		Supportive			Colchicine			Budesonide		
		Admission	Discharge	*P* value	Admission	Discharge	*P* value	Admission	Discharge	*P* value

Admission SpO2 + O2 supp vs discharge SpO2(mean)	Sat w/cannula	94	94.5	0.65	95	95.2	0.85	92	92.4	0.91
	Sat w/face mask	95	99	0.25	95.3	95.3	0.85	89.3	93.3	0.82
	Sat w/non-rebreather mask	91.4	94.4	0.17	88.8	94.9	0.08	91.6	94.2	0.51
Admission PiO2/FiO2 ratio vs discharge PiO2/FiO2 ratio (mean)		102	221	≤0.016	118.4	200	≤0.001	80	217	≤0.01

**Table 4:** Outcomes, hospitalization, and O2 supplementation time between admission and discharge.

Study outcomes		Supportive	Colchicine	Budesonide	*P* value
Time of hospitalization (mean)		10 (6–14)	8 (5–12)	8 (5–12)	0.60
Time on O2 supp from admission to cure (mean)		20 (12–29)	19 (10–29)	20 (12–29)	0.97
Outcome	ICU/death	7	3	5	0.67
	Discharge w/readmission	1	3	2	0.32
	Discharge w/cure	13	8	7	0.78

## Data Availability

Data are available on request to the corresponding author.
